# Correction for Wang et al., “An inhibitor/anti-inhibitor system controls the activity of lytic transglycosylase MltF in *Pseudomonas aeruginosa*”

**DOI:** 10.1128/mbio.03382-23

**Published:** 2024-02-02

**Authors:** Michelle Wang, Sheya Xiao Ma, Andrew J. Darwin

## AUTHOR CORRECTION

Volume 14, no. 6, e02022-23, 2023, https://doi.org/10.1128/mbio.02022-23. We chose the term peptidoglycan hydrolysis to refer collectively to the process of peptidoglycan lysis but inadvertently continued to use similar words when referring to MltF specifically. Lytic transglycosylases can participate in the collective breakdown of peptidoglycan, which does involve hydrolysis reactions, and structurally they fall into the glycoside hydrolase family. However, we are aware that these enzymes themselves do not use water during catalysis. Therefore, to improve accuracy, collective terms referring to peptidoglycan/cell wall hydrolase(s), hydrolysis, and hydrolyzed should instead be lytic enzyme(s), lysis, and lysed. The same corrections are made for specific references to MltF activity. These wording changes do not alter any findings or conclusions, which do not depend on the chemical catalytic mechanism of MltF.

