# Supplementary material for: An inhibitor/anti-inhibitor system controls the activity of lytic transglycosylase MltF in Pseudomonas aeruginosa
Source: mBio. 2023 Dec 4;14(6):e02022-23. doi: 10.1128/mbio.02022-23 (PMC10746161; doi:10.1128/mbio.02022-23)
Supplement: Figure S1 — Complementation of ΔPA3978 mutant XcpQ sensitivity. [file mbio.02022-23-s0001.pdf]

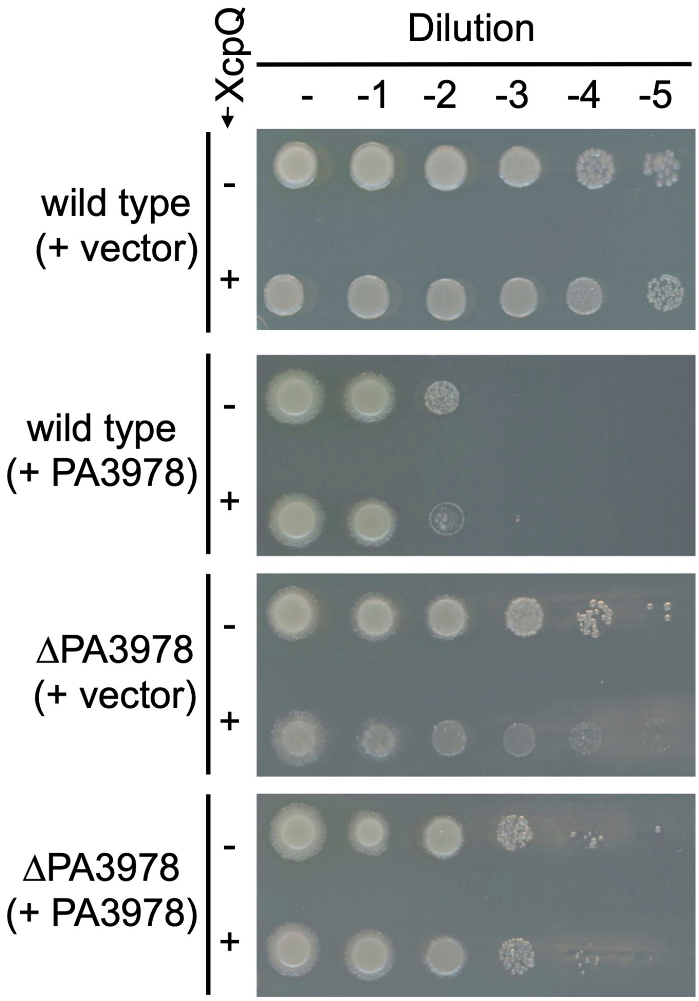


**Supplementary Figure S1** Complementation of ΔPA3978 mutant XcpQ-sensitivity. Wild type and ΔPA3978 strains contained plasmid pAJD2287 encoding PA3978 expressed from its native promoter, or the empty vector control. Strains also contained either the *tac* promoter expression plasmid pVLT35 (- XcpQ), or the *xcpQ*$^{+}$ derivative pAJD942 (+XcpQ). Serial dilutions of normalized saturated cultures were spotted onto LB agar containing 125 μM IPTG and incubated at 37˚C for approximately 24 h. Plasmid pAJD2287 was toxic, especially in the wild type strain that also had endogenous PA3978. Therefore, XcpQ production was induced with a lower IPTG concentration in this experiment to reduce the overall amount of stress.
