# Supplementary material for: An inhibitor/anti-inhibitor system controls the activity of lytic transglycosylase MltF in Pseudomonas aeruginosa
Source: mBio. 2023 Dec 4;14(6):e02022-23. doi: 10.1128/mbio.02022-23 (PMC10746161; doi:10.1128/mbio.02022-23)
Supplement: Table S1 — Known or predicted cell envelope proteins that co-purified in each of three PA3978-FLAG pulldowns with an average PSM ≥ 10. [file mbio.02022-23-s0002.pdf]

Supplemental Table S1: Known or predicted cell envelope proteins that co-purified in each of three PA3978-FLAG pulldowns with an average PSM ≥ 10

		PA3978-FLAG	PA3978 control	PA3978-FLAG Experiment 1		PA3978-FLAG Experiment 2		PA3978-FLAG Experiment 3		PA3978 control Experiment 1		PA3978 control Experiment 2		PA3978 control Experiment 3	
Accession	Protein	Average PSM	Average PSM	Coverage #1	PSM #1	Coverage #2	PSM #2	Coverage #3	PSM #3	Coverage D2	# PSM Control_1	Coverage E2	# PSM Control_2	Coverage F2	# PSM Control_3
Q9HXN1	MltF	375	9	75.71	434	52.24	332	75.71	359	39.18	26	5.10	2	0.00	0
G3XD26	PA3978 (Bait)	371	10	46.15	428	46.15	275	46.15	409	35.71	26	18.68	3	0.00	0
Q9HT71	PA5502	90	3	38.17	77	30.15	80	38.17	114	14.89	7	14.50	3	0.00	0
P34750	PilQ	40	25	44.40	40	40.62	38	49.44	43	24.65	20	44.40	39	19.61	15
G3XD74	DacC	39	21	51.30	62	24.87	23	51.30	33	35.23	21	47.41	32	34.46	11
P24474	NirS	32	30	52.11	35	22.36	19	56.51	42	34.86	32	46.13	34	34.68	23
P13794	OprF	31	26	43.71	32	40.00	36	43.71	26	44.00	27	46.00	44	14.86	7
Q9HXR3	PA3729	31	19	39.10	31	21.51	22	39.10	39	18.60	15	26.45	20	26.16	22
Q9I1I5	Gcd	30	16	52.43	50	3.74	2	46.58	38	17.06	12	21.17	14	28.39	21
Q51487	OprM	30	13	54.02	45	22.89	14	52.16	30	24.74	13	31.34	15	25.36	12
Q9I2T8	PpiD	28	22	55.88	47	17.39	12	41.22	25	38.16	27	43.00	33	9.02	6
G3XD28	PilM	26	21	58.76	29	23.45	12	52.54	37	41.53	18	53.67	28	37.85	17
Q9I3G6	PA1551	26	13	28.03	24	16.77	24	24.20	29	11.04	7	24.20	16	19.32	15
Q9I3G2	CcoP2	26	19	55.19	36	19.16	10	44.81	31	29.22	13	44.16	24	43.18	19
P32722	OprD	25	27	21.90	20	17.61	25	25.73	29	17.61	20	18.51	20	25.73	40
Q9I083	PA2760	24	24	37.41	24	31.06	24	39.76	24	29.18	24	31.53	23	38.59	26
Q9HVT2	PA4489	23	7	29.75	39	4.09	5	17.81	25	8.44	9	10.49	10	2.04	2
P21175	BraC	23	34	53.35	16	38.07	21	56.84	32	42.90	28	58.98	58	42.36	15
Q9HUA6	OpgH	23	4	28.46	27	16.84	18	24.16	23	6.50	5	3.72	3	7.55	5
Q9HUM2	HflK	23	19	55.75	32	34.75	14	44.25	22	21.50	9	44.00	31	47.50	18
Q9HV48	FtsH	22	11	47.10	48	4.85	3	25.98	15	11.11	8	18.47	12	22.54	13
P52477	MexA	22	13	72.06	44	19.84	8	23.50	13	21.67	10	43.34	19	24.54	9
G3XDA5	OprE	21	17	34.13	16	30.43	25	46.96	22	22.61	13	29.13	20	34.57	18
G3XD11	OprH	21	27	52.50	23	52.50	17	53.00	22	52.50	15	61.00	42	64.00	25
Q9HVK2	PA4582	20	22	48.29	21	29.66	13	51.18	26	35.43	19	34.91	18	58.01	29
Q9HXU8	LptF	19	17	52.87	25	40.23	20	39.46	12	29.12	6	48.28	26	52.87	20
Q9HXI1	DecD	19	16	26.94	17	20.81	18	27.10	21	24.84	19	28.06	21	15.16	8
Q9HU38	PA5146	18	11	26.53	18	19.87	15	28.93	22	18.00	12	21.60	13	8.80	7
P14532	CcpA	18	11	28.32	21	18.21	17	35.26	17	18.21	10	23.41	12	32.08	12
Q9HW93	PctC	18	11	34.97	20	15.82	12	38.29	21	21.36	13	26.58	15	10.44	5
G3XD24	PctA	17	12	29.73	19	17.33	13	33.86	20	19.71	13	30.21	18	12.08	6
Q9I2T4	PA1810	17	1	11.87	4	25.37	21	40.49	26	4.23	2	0.00	0	0.00	0
Q9HV64	PA4735	17	3	24.36	23	10.02	9	20.22	18	7.08	6	2.30	2	0.00	0
Q9HTJ2	BetA	17	9	49.02	25	3.57	2	47.59	23	17.11	8	30.66	11	21.39	9
Q9HW91	PctB	17	14	29.25	19	15.10	13	35.29	18	19.71	14	32.43	21	14.15	7
Q9HT53	PA5520	17	38	53.44	17	14.57	3	65.18	30	45.34	20	53.44	31	68.42	63
Q9HUK6	FimX	16	3	16.93	11	11.58	8	35.89	30	3.47	2	6.22	4	2.75	2
Q9I5Z9	PA0537	16	9	67.33	19	34.65	6	63.86	24	44.55	10	44.55	9	48.02	8
Q9HU49	PA5135	16	0	42.02	8	34.24	20	66.93	20	0.00	0	0.00	0	0.00	0
P24735	AmpC	16	8	35.52	13	26.45	12	45.84	22	17.63	8	25.44	11	18.89	6
Q9HUB8	UbiB	15	5	30.02	16	17.26	12	30.77	18	6.94	5	7.50	5	6.75	4
Q9HZ12	PA3228	15	6	23.77	16	19.84	13	24.10	15	10.00	8	4.59	2	14.43	9
Q9HZA6	FimV	14	14	25.57	20	10.99	10	17.41	13	17.19	12	23.07	15	20.67	16
Q9I402	PA1342	14	22	26.82	6	31.46	13	60.93	24	39.40	23	67.22	35	17.88	7
Q9HWF5	SecY	14	4	22.40	13	13.57	11	24.89	18	8.37	4	8.14	5	12.67	4
Q9I7A7	PA0020	14	13	26.39	10	34.60	14	46.92	18	33.43	16	42.82	20	14.96	4
Q9HW32	IcmP	14	18	44.39	19	25.34	10	30.04	13	20.63	12	47.98	28	34.30	13
Q9HT06	YidC	14	8	23.53	14	14.19	12	23.53	15	12.11	6	17.65	12	12.11	7
P72151	FliC	13	13	9.02	15	3.89	5	6.97	20	0.00	0	9.02	27	9.02	13
P42257	PilJ	13	20	30.21	17	7.04	4	27.57	19	21.26	22	30.21	31	13.93	7
P50601	TolB	12	18	18.98	8	42.82	18	29.86	11	43.29	20	62.27	28	15.51	6
Q9I056	Cpg2	12	0	10.19	4	27.18	21	30.34	11	0.00	0	0.00	0	0.00	0
Q9I407	AnsB	12	20	25.97	9	18.51	8	49.72	18	31.77	20	46.13	28	32.04	11
Q9HZ86	WbpM	11	3	25.86	15	5.41	3	25.11	15	3.61	2	6.62	3	8.27	4
Q9I522	LpxO2	11	4	35.58	14	11.54	6	34.62	13	11.22	3	10.58	4	16.99	5
Q9HTT8	PA5257	11	6	26.21	13	14.08	6	33.74	14	16.75	6	22.82	10	5.83	2
Q9HWW1	OprG	11	11	27.16	10	18.53	12	26.29	11	18.53	5	26.29	17	34.48	12
Q9HYX0	PA3271	11	5	12.25	13	1.81	2	14.84	17	0.00	0	1.98	2	11.22	14
Q9HVS0	OpdP	11	10	20.87	10	12.81	8	26.45	14	11.16	6	20.87	11	26.24	13
Q9HUD4	PA5037	10	6	25.95	15	9.98	7	14.70	9	7.99	5	17.42	8	11.25	4
Q9HVY6	PA4429	10	8	40.38	10	21.54	10	36.92	11	31.15	8	40.38	13	16.54	4
Q9HTT7	PA5258	10	6	49.20	14	13.56	5	43.09	12	26.86	8	24.20	9	0.00	0
Q9HVR9	PA4502	10	16	21.66	8	5.08	5	32.02	18	30.32	20	35.40	24	11.11	5
Q9HTV9	PA5231	10	2	8.73	8	7.10	7	18.12	15	0.00	0	2.73	2	5.35	4
P33641	BamD	10	9	46.92	19	12.32	3	30.50	8	19.06	8	40.18	14	18.18	4
Q9I5U3	SurA	10	12	20.14	7	13.91	5	46.28	18	30.46	16	45.32	17	5.04	2
Q9HVS5	PA4496	10	10	10.43	5	13.04	11	21.97	14	18.62	14	16.39	16	0.00	0
